# Monocyte-to-HDL-cholesterol ratio is associated with Ascending Aorta Dilatation in Patients with Bicuspid Aortic Valve

**DOI:** 10.4314/ahs.v21i1.14

**Published:** 2021-03

**Authors:** Burak Acar, Cagrı Yayla, Murat Gul, Mustafa Karanfil, Sefa Unal, Fatih Uçar, Serdar Mevlut Kuyumcu, Ahmet Goktug Ertem, Yasin Ozen, Mustafa Bilal Ozbay, Ozcan Ozeke, Sinan Aydogdu

**Affiliations:** 1 Department of Cardiology, Faculty of Medicine, Kocaeli University, Kocaeli/Turkey; 2 Department of Cardiology, Yuksek Ihtisas Education and Research Hospital, Ankara/Turkey; 3 Department of Cardiology, Aksaray University, Aksaray/Turkey; 4 Department of Cardiology, Trakya University, Edirne/Turkey; 5 Department of Cardiology, Suleyman Demirel University, Isparta/Turkey

**Keywords:** Bicuspid aorta, aorta aneurysm, monocyte HDL ratio, inflammation

## Abstract

**Background:**

The importance of monocyte count-to-HDL-cholesterol ratio (MHR) in cardio- vascular diseases has been shown in various studies. Ascending aortic dilatation (AAD) is a common complication in the patients with bicuspid aortic valve. In this study, we aimed to investigate the relationship between MHR and the presence of aortic dilatation in the patients with bicuspid aortic valve.

**Methods:**

The study population included totally 347 patients with bicuspid aortic valve.169 patients with aortic dilatation (ascending aorta diameter ≥ 4.0 cm) and 178 patients with no aortic dilatation. Echocardiographic and laboratory measurement was done and compared between groups.

**Results:**

The mean age of the participants was 44.7 ± 15.4 years and average ascending aorta diameter was 3.2 ± 0.3 cm in dilatation negative group and 4.4 ± 0.4 cm in positive group. MHR was significantly increased in in patients with aortic dilatation. MHR and uric acid level was independently associated with the presence of aortic dilatation in the patients with bicuspid aortic valve.

**Conclusion:**

We found a significant relationship between MHR and aortic dilatation in the patients with bicuspid aortic valve.

## Introduction

One of the most common congenital cardiac malformation in clinical practice is bicuspid aortic valve (BAV). Some of the clinically important complications usually related to BAV are aortic valve dysfunction, aortic dissection, infective endocarditis, and ascending aorta dilatation (AAD)^1^. AAD causes more problems compared to the others because it is included in the process of aortic regurgitation in BAV^2^ as well as increasing aortic stiffness^3^. There is also the risk of aortic dissection^4^ where aortic surgery is needed^5^. For many years there have been a lot of discussion about the causes of aortic dilatation. Possible grounds leading to aortic dilatation are anomalous blood flow in the ascending aorta produced by the anomalous dynamics of BAV^6^ and genetic causes^7^. Hemodynamic disturbances of the BAV, including abnormal flow turbulence, poststenotic dilatation, and increased stroke volumes of aortic insufficiency are believed to be the most common cause for aneurysmal dilatation of the aortic root and ascending aorta^8^. It has been also claimed that eccentric flow patterns due to abnormal valve may lead to a differential distribution of aortic wall shear stress and subsequent flow-induced vascular remodeling of the aortic wall^9^. It is unfortunate that the physiopathology of AAD in BAV patients is yet to be discovered, which could be a result of other processes' potential involvement.

It is widely established that normal HDL regulates cholesterol efflux from tissues and modulates inflammation and oxidative stress as a successful anti-inflammatory and antioxidant molecule^10^ and its major protein component, apolipoprotein A-I (apo A-I), show an anti-inflammatory effect on human monocytes by inhibiting activation of CD11b. Bicuspid aorta patients with aortic dilatation have lower HDL cholesterol levels than the patients with no aortic dilatation^11^.

Circulating monocytes as an origin of various cytokines and molecules, interact with platelets and endothelial cells and leading destruction of extracellular matrix by proteases, medial destruction connected with apoptosis or differentiation of smooth muscle cells, increased oxidative stress, neovascularization and thrombus formation^12^. Also, studies prove that cytokines which have been released from monocytes play a role in bicuspid valve degeneration^13^.

Recently monocyte count-to-HDL-cholesterol ratio (MHR) has emerged as a new predictor of cardiovascular events in chronic kidney disease^14^. It has also been shown that MHR is associated with the slow coronary flow^15^, stent thrombosis^16^, coronary artery ectasia^17^ impaired aortic elastic properties in hypertensives^18^ and aneurysm size in asymptomatic abdominal aorta aneurysm patients^19^.

Monocytes and HDL-cholesterol are separately linked to degeneration pathophysiology of the bicuspid valve and there is a necessity to improve the present-day diagnosis and management of bicuspid valve illness, that's why we sought to identify the relation of admission MHR with the maximum diameter of the climbing aorta in bicuspid aortic valve patients.

## Methods

### Study population

Between October 2013 and December 2016, a total of 362 patients with bicuspid aortic valve were screened who admitted to our outpatient clinic. 15 patients were excluded due to severe valvular dysfunction (9 patients with severe aortic regurgitation and 6 patients with severe aortic stenosis). Finally, 347 patients enrolled in this study. Data was obtained from the medical records. The patients with bicuspid aorta were divided into two groups according to ascending aortic diameter (ascending aorta < 4.0 or ≥ 4.0 cm). The study group was also included aortic ectasia or also patient with aneurysmal dilatation (e.g >5 or 5.5 cm). All participants were evaluated by echocardiography. Patients with severe valvular heart disease history of rheumatic fever, and prosthetic valves, heart failure, malignancy, connective tissue disorder, renal or hepatic dysfunction, acute or chronic infection or inflammatory condition, hematologic diseases including anemia, chronic obstructive pulmonary disease and unavailable medical records were excluded. Baseline demographic and clinical characteristics were reviewed. Aortic stenosis was defined as aortic jet velocity >2.5 m/s and severe AS was defined as mean gradient >40 mmHg, aortic valve area (AVA) <1 cm2 and peak aortic jet velocity >4.0 m/s. Severe aortic regurgitation defined by a jet height to LV outflow tract dimension ratio of 0.6 or a prominent holodiastolic flow reversal in the aortic arch or the abdominal aorta. In the presence of both of valvular dysfunction (AS and AR) defined as mixed dysfunction.

Hypertension was defined as documentation of a systolic blood pressure of ≥140 mmHg and/or a diastolic blood pressure of ≥90 mmHg in at least two measurements or active use of any antihypertensive agent. Diabetes mellitus was diagnosed as a fasting plasma glucose level over 126 mg/dl or glucose level over 200 mg/dl at any measurement or active use of an antidiabetic agent. Smoking was defined as if the patient is a current smoker or not. Dyslipidemia was defined as active use of lipid-lowering agent or LDL level > 100 at any measurement. The study was in compliance with the principles outlined in the Declaration of Helsinki and approved by local institutional review board committee (Yuksek Ihtisas Education and Research Hospital, Date 22/11/2017, Number 11915).

### Transthoracic echocardiography

Echocardiographic evaluation was performed by using a VIVID 7 Dimension Cardiovascular Ultrasound System (Vingmed-General Electric, Horten, Norway) with a 3.5 MHz transducer. Echocardiographic examination was performed in the left lateral decubitus position. Parasternal long- and short-axis views and apical views were used as standard imaging windows. Ejection fraction was calculated by using modified Simpson method. Two-dimensional measurements of the aortic root were made at end- diastole in the parasternal long-axis views using the leading edge to leading edge technique in the views showing the largest aortic diameters at three levels as: (1) annulus, (2) sinus of Valsalva and (3) proximal ascending aorta. The maximal diameter was evaluated in this study. End systolic and end diastolic dimensions were measured from parasternal long-axis view. Maximal and mean aortic gradient was calculated by Doppler echocardiography. All echocardiographic examinations were performed by an experienced cardiologist. All measurements were calculated according to the criteria proposed by the American Society of Echocardiography^20^.

### Laboratory measurements

Blood samples were obtained from the antecubital vein after a fasting period of 12 hours. Coulter Counter LH Series (Beckman coulter Inc, Hialeah, Florida) was used for complete blood count (CBC) and C reactive protein levels (CRP). Plasma levels of triglyceride, high-density lipoprotein, low-density lipoprotein, glucose, creatinine, uric acid was evaluated using an automated chemistry analyzer (Abbott Aeroset, USA) using commercially available kits (Abbott, USA). MHR was calculated as monocyte counts divided by those of HDL level and using the same blood samples drawn.

### Statistical analysis

All statistical analysis was performed in SPSS 20.0 Statistical Package Program for Windows (SPSS Inc., IL, USA). The normality of distributions of the parameters was assessed by the Kolmogorov-Smirnov test. Quantitative variables with a normal distribution were specified as the mean + standard deviation and those with non-normal distribution were specified with median (interquartile range or percentile); categorical variables were specified with number and percentage values. Continuous variables were compared using the independent samples t test for normally distributed variables, and the Mann-Whitney U test when the distribution was skewed. The chi-square test or Fisher's exact test was used to compare categorical variables. Univariate logistic regression analysis was used to assess the relationship between variables and aortic dilatation. Variables found to be p value of < 0.1 in the univariate analysis were then used in forward stepwise multivariate logistic regression analysis in order to determine independent predictors of aortic dilatation. The results of the regression analyses were presented as odd ratios (OR) and 95% confidence intervals (CI). A p value <0.05 was considered statistically significant.

## Results

Clinical and demographical characteristics of study groups are shown in Table 1. The mean age of the participants was 44.7 ± 15.4 years and 65 % of patients were male. There was no statistically significant difference among the groups by means of gender, rate of hypertension, diabetes mellitus, smoking or hyperlipidemia. The patients with aortic dilatation were older than the dilatation negative group (47 ± 15.8 vs.42 ± 14.7, p=0.001). Echocardiographic measurements were not different between the groups except aortic diameter and aortic regurgitation ratios. Average ascending aorta diameter was 3.2 ± 0.3 cm in dilatation negative group and 4.4 ± 0.4 cm in positive group. Aortic regurgitation ratio was significantly higher in dilatation positive group than negative group (90.5 % vs. 82.6%, p=0.041). Aortic stenosis and mixed dysfunction was not differed between the groups (Table 1).

**Table 1:** Baseline clinical and echocardiographic characteristics of the patients according to aorta dilatation

	Group I Dilatation (-) (n=178)	Group II Dilatation (+) (n=169)	P Value
Male, n (%)	(115) (64.6%)	(111) (65.7%)	0.830
Age, years	42 ± 14.7	47 ± 15.8	**0.001**
Hypertension, n (%)	55 (31%)	66 (39)	0.141
Diabetes, n (%)	16 (9%)	22 (13%)	0.232
Hyperlipidemia, n (%)	31 (17%)	29 (17%)	0.523
Smoking, n (%)	30 (25%)	49 (36%)	0.135
Ejection Fraction (%)	60 ± 6.2	59±6.3	0.846
Left ventricular end- diastolic diameter (mm)	47 ± 5.3	49 ± 5.3	0.277
Left ventricular end- systolic diameter (mm)	31 ± 5.4	33 ± 4.6	0.129
Maximal Aortic Gradient (mmHg)	26 (4–110)	31 (5–138)	0.145
Mean. Aortic Gradient (mmHg)	15 (2–56)	18 (2–90)	0.082
Average ascending aorta diameter (cm)	3.2 ± 0.3	4.4 ± 0.4	**<0.001**
Aortic insufficiency	147 (82.6%)	153 (90.5%)	**0.041**
Aortic stenosis	69 (38.8%)	79 (46.7%)	0.158
Mixed dysfunction	62 (34.8%)	75 (44.4%)	0.791

*Data are expressed as the median (25th percentile- 75th percentile)) for not normally distributed and mean ± standard deviation or frequency for other factors. Significant difference if p<0.05

The comparison of laboratory parameters is shown in Table 2. Monocyte (0.67 ± 0.40 vs. 0.55 ± 0.19, p=0.001), MHR (15.0 ± 10.3 vs. 13.0 ± 5.9, p<0.001), CRP (10.1 ± 27.8 vs. 5.3 ±11.4 mg/L; p=0.03), uric acid (5.2 ± 1.77 vs. 4.5 ± 1.62, p=0.001) and creatinine (0.98 ± 0.24 vs. 0.92 ± 0.23, p=0.02) levels were significantly higher in the dilatation group compared to the dilatation negative group. However, glucose, hemoglobin, WBC, platelet and cholesterol levels, was not significantly different between the groups.

**Table 2:** Comparison of patients with ascending aorta dilatation and control individuals in terms of biochemical and hematological characteristics

	Group I Dilatation (-) (n=178)	Group II Dilatation (+) (n=169)	P Value
Glucose, mg/dL	102 ± 46.1	100 ± 24.3	0.645
Creatinine, mg/dL	0.92 ± 0.23	0.98 ± 0.24	**0.028**
LDL, mg/dL	106 ± 37	112 ± 30	0.094
HDL, mg/dL	45 ± 12	45 ± 11	0.824
Hemoglobin, g/dL	14 ± 1.6	13 ± 1.7	0.421
Platelet, x10^3^/L	242 ± 61	241 ± 62	0.833
WBC, x109/L	7.7 ± 2.1	7.5 ± 2.0	0.327
Monocyte, x109/L	0.55 ± 0.19	0.67 ± 0.40	**0.001**
Monocyte/ HDL Ratio	13.0 ± 5.9	15.0 ± 10.3	**<0.001**
Uric acid	4.5 ± 1.62	5.2 ± 1.77	**0.001**
C-reactive protein	5.3 ±11.4	10.1 ± 27.8	**0.032**

*LDL, low density lipoprotein; HDL, high density lipoprotein; WBC, white blood cell.

*Data are expressed as the median (25th percentile- 75th percentile)) for not normally distributed and mean ± standard deviation or frequency for other factors. Significant difference if p<0.05.

Assessment of the MHR and other most important valuables with each type of valve dysfunction in patients was shown in Table 3. CRP levels were significantly higher in patients with aortic stenosis and mixed dysfunction. There was no other significant difference between the groups.

**Table 3:** Assessment of the parameters with each type of valve dysfunction

	Aortic stenosis			Aortic Insufficiency			Mixed Dysfunction		

	(+)(n=148)	(-)(n=299)	P value	(+)(n=300)	(-)(n=47)	P value	(+)(n=137)	(-)(n=210)	P value
**MHR**	13.3 ± 6.7	12.3±5.1	0.115	12.8±6.0	12.0±4.3	0.397	13.4±6.8	12.3±5.1	0.104
**Uric** **acid**	4.7±1.6	4.9±1.77	0.418	4.8±1.7	4.9±1.8	0.596	4.8±1.66	4.9±1.76	0.585
**CRP**	11.4 ± 9.4	4.74 ±6.8	**0.005**	8.2±6.4	3.3±3.2	0.152	11.9±4.76	4.76±9.55	**0.003**

*MHR, monocyte/HDL ratio; CRP, C reactive protein; Mixed Dysfunction, presence of both of the aortic stenosis and aortic insufficiency.

The association of aortic dilatation with the clinical and laboratory parameters evaluated in univariate and stepwise multivariate linear regression analysis was shown in Table 4. In multivariate logistic regression analysis, higher MHR level was found significantly and independently associated with the presence of aortic dilatation (OR: 1.048; 95% CI: 1.003–1.095; p = 0.037). Moreover, uric acid levels (OR: 1.252; 95% CI: 1.037–1.512; p = 0.019) was also independently associated with the presence of aortic dilatation.

**Table 4:** Independent predictors of aorta dilatation in patients with bicuspid aortic valve

	*Univariate*			*Multivariate*		

	OR	CI 95 %	p-value	OR	CI 95 %	p-value
Age	1.023	1.009–1.038	0.001	1.011	0.992–1.030	0.268
Smoking	1.690	0.991–2.882	0.054	1.581	1.094–3.589	0.064
Creatinine	2.858	1.131–7.222	0.026	1.962	0.607–6.340	0.260
Uric acid	1.247	1.098–1.415	0.001	1.252	1.037–1.512	**0.019**
CRP	1.029	1.000–1.058	0.052	1.013	0.988–1.039	0.303
MHR	1.051	1.031–1.099	<0.001	1.048	1.003–1.095	**0.037**

CRP, C reactive protein; MHR, monocyte-high density lipoprotein ratio. Bold means presence of significance (p<0.05)

## Discussion

This study supposes that increased monocyte count to HDL-cholesterol ratio is independently associated with a maximum diameter of the ascending aorta in BAV patients. Moreover, uric acid level is related with aortic dilatation in the patients with BAV.

BAV is the most popular congenital heart defect which affects 1.3% of the population^21^,^22^. Patients with a BAV present with variable clinical scenarios. Some cases may be completely asymptomatic, however, approximately 30% of individuals with a BAV will show complications^23^. The most common complication of BAV is valve dysfunction^24^, however, the most important effect of BAV is dilatation of ascending aorta^25^, ^26^. The disease is often progressive and might cause catastrophic clinical events such as aortic rupture and dissection. One in 100 BAV patients per year will experience an ascending aortic aneurysm, with a chance of dissection possibly 8 times higher in this group^27^. Nonetheless, it remains unclear what causes BAV aortopathy. A genetic predisposition may lead to diffuse aortopathy^28^. Furthermore, cusp fusion and abnormal BAV morphology can trigger abnormal regional blood flow patterns in the ascending aorta and result in discrete and localized areas of illness. The possibility of both structures' coexistence remains solid and they also work synergistically to cause heterogeneous patterns of aortic dilation that is often seen in patients with BAV^29^.

Inflammation may be related to aortopathy^30^. Kasapkara et al. found that neutrophil-lymphocyte ratio, one of the major inflammation marker, is related to higher ascending aorta diameter in BAV patients^31^. Monocytes as a distinct type of leukocytes are the key player in this process. Activated monocytes interact with damaged or activated endothelium, which results in overexpression of proinflammatory cytokines/adhesion molecules including monocyte chemotactic protein 1 ligand and vascular cell adhesion molecule 1 and intercellular adhesion molecule 1. Thereafter, monocytes differentiate into the macrophages that ingest oxidized LDL cholesterol and form the dangerous foamy cells^32^. Furthermore, HDL molecules counteract the migration of macrophages and promote efflux of oxidized cholesterol from these cells. Recent studies also indicate a role of HDL in controlling of monocyte activation, adhesiveness, and inflammation^33^ and in controlling the proliferation of progenitor cells that give rise to monocytes^34^. Besides its anti-inflammatory and antioxidative effects, HDL molecules also enhance vasorelaxation and increased endothelial nitric oxide synthase expression^35^, ^36^. Therefore, monocytes exert a proinflammatory and pro-oxidant effects, but HDL-C functions as a reversal factor during those processes

One of the important underlying mechanisms in the pathophysiology of aortopathy in BAV patients is oxidative stress^37^. Billaud et al show that elevated superoxide-anion levels in ascending aortic tissue from patients with BAV without an aneurysm compared with tissue from patients with BAV with an aneurysm, or patients with a normal trileaflet aortic valve in the presence or absence of disease. Importantly, BAV tissues failed to show elevated superoxide-dismutase activity, a protective enzyme that functions to scavenge superoxide-anions and convert them to the less-reactive intermediate, hydrogen peroxide^38^. There was a noted increase in peroxidase activity, capable of clearing the hydrogen peroxide; however, the investigators also identified increased oxidative damage, as measured by an increase in 8-iso-prostaglandin-F2-alpha, in all BAV aortic specimens compared with aortic specimens from patients with trileaflet aortic valve. The main source of oxygen radicals in the vein wall is monocytes^39^,^40^. HDL has been demonstrated to possess significant antioxidant activity that is primarily mediated via the inhibition of the oxidation of LDL with a subsequent reduction of the cellular uptake by the monocyte-macrophage system^41^. The mechanism by which HDL performs an antioxidant activity is complex and multifactorial. Transition metals have played a role in oxidative stress and HDL has been demonstrated to exhibit chelation properties due to be a presence of proteins such as ceruloplasmin on the surface of the lipoprotein, although the clinical relevance is controversial^42^. Lipid peroxidation products are derived from oxidized LDL and have been demonstrated to be cytotoxic and predispose to atherosclerosis. HDL has been demonstrated to accept hydroperoxides from oxidized membranes in vitro studies, which would potentially provide a pathway for excretion or detoxification^43^. For this reason, the balance between monocyte amount and HDL may explain the effect of oxidative stress in aortopathy in BAV patients.

In this study, we also found that uric acid was independent predictor of aortic dilatation. Uric acid (urate, UA), the final oxidation product of purine catabolism in humans, is considered to be a powerful predictor of cardiovascular risk and poor outcome^44^. Tang et al. have showed that uric acid levels was associated with aortic root dilatation in hypertensive patients^45^.

## Limitations

There are some limitations of this study. First, it was a retrospective study. We could not evaluate the age- and body size- adjusted nomograms for aortic aneurysm. Using a spot laboratory value rather than values at a time interval is another limitation of this study. In addition, we did not evaluate other cytokines or inflammatory markers such as fibrinogen were not used. To clarify this hypothesis, multicenter, large-scale, randomized and prospective studies are needed.

## Conclusion

We demonstrated for the first time that combination of high circulating monocyte count and low HDL-cholesterol concentration is independently associated with ascending aorta aneurysm size in BAV patients. MHR may be simply calculated from the complete blood count analysis and this parameter may be used for risk stratification in patients with BAV.
